# Size-Controllable Synthesis of Monodisperse Magnetite Microparticles Leading to Magnetically Tunable Colloidal Crystals

**DOI:** 10.3390/ma15144943

**Published:** 2022-07-15

**Authors:** Toya Seki, Yutaro Seki, Naoto Iwata, Seiichi Furumi

**Affiliations:** Department of Chemistry, Graduate School of Science, Tokyo University of Science, 1-3 Kagurazaka, Shinjuku, Tokyo 162-8601, Japan; 1322578@ed.tus.ac.jp (T.S.); 1320568@alumni.tus.ac.jp (Y.S.); n-iwata@rs.tus.ac.jp (N.I.)

**Keywords:** colloidal crystals, magnetite, microparticles, Bragg reflection, magnetic response

## Abstract

Colloidal crystals (CCs) are periodic arrays of monodisperse microparticles. Such CCs are very attractive as they can be potentially applicable as versatile photonic devices such as reflective displays, sensors, lasers, and so forth. In this article, we describe a promising methodology for synthesizing monodisperse magnetite microparticles whose diameters are controllable in the range of 100–200 nm only by adjusting the base concentration of the reaction solution. Moreover, monodisperse magnetite microparticles in aqueous suspensions spontaneously form the CC structures under an external magnetic field, leading to the appearance of Bragg reflection colors. The reflection peak can be blue-shifted from 730 nm to 570 nm by the increase in the external magnetic field from 28 mT to 220 mT. Moreover, the reflection properties of CCs in suspension depend on the microparticle concentration in suspension and the diameter of the magnetite microparticles. Both fine-control of microparticle diameter and investigation of magneto-optical properties of CCs would contribute to the technological developments in full-color reflective displays and sensors by utilizing these monodisperse magnetite microparticles.

## 1. Introduction

Colloidal crystals (CCs) are periodic arrays of monodisperse microparticles [1,2,3,4,5]. Such CCs can be regarded as one of the three-dimensional (3D) photonic crystals that show the forbidden regions for photons in the dispersion spectrum, that is, the photonic bandgaps (PBGs), due to the spatial periodicity of the refractive indices between the colloidal microparticles and dispersion media [6,7,8]. One of the most important properties of CCs is their ability to show visible reflection using colloidal microparticles with diameters of several hundred nanometers. The reflection peak wavelength (*λ*) of CCs is approximately calculated according to the following Bragg’s equation [8,9].

(1) λ=2ndsinθ

where *n* is the effective refractive index of materials, *d* is the interparticle spacing of CC, and *θ* is the angle of incident light, that is, the Bragg angle. When the value of *λ* is comparable to the wavelength range of visible light, the PBGs of CCs can be observed as Bragg reflection colors. Introduction of stimuli-responsive materials in the microparticles and/or the background encompassing them ensures the on-demand control of reflection peaks of CCs by external stimuli such as temperature, ionic strength, mechanical force, and so forth [10,11,12,13]. Owing to this unique optical property, the CCs are very attractive as they can be potentially applicable in versatile photonic devices as color reflective displays, sensors, lasers, and so forth.

Previously, some types of methods for preparing monodisperse magnetite microparticles have been reported [14]. These microparticles can be applied to CCs, which can be categorized into two types: 3D or one-dimensional (1D) CCs [15,16,17,18,19]. For instance, Asher and co-workers reported that highly charged monodisperse polystyrene (PS) microparticles encapsulating superparamagnetic nanoparticles can self-assemble into the 3D CC structures due to their highly charged surface [19]. The attractive force between microparticles induced by applying an external magnetic field was not strong because of the low content of the magnetic component in the microparticles, leading to the limited tuning range of Bragg reflection wavelength and insufficient magnetic responsivity of the CCs. On the other hand, Yin and co-workers developed an intriguing strategy for synthesizing polyelectrolyte-capped monodisperse superparamagnetic magnetite microparticles that can be directly used as the building blocks of the 1D CC structures [17]. These magnetite microparticles are advantageous in the following three aspects. First, they can show higher saturated magnetizations and superior magnetic responsivity because they only consist of magnetite. Second, the magnetite microparticles can be easily dispersed in water due to their highly negatively charged surface offered by polyelectrolyte surfactants. Finally, magnetite microparticle diameters can be controlled in the range between 30 nm and 180 nm with narrow size distribution. By utilizing these characteristics, the magnetite microparticles in aqueous suspensions spontaneously form the 1D chain-like CC structures under an external magnetic field, leading to the appearance of Bragg reflection colors due to the spatial modulation of the refractive indices between magnetite microparticles and water as a dispersion medium. It has been considered that the 1D chain-like CC structure can be formed by the balance of attractive force induced by magnetic dipole–dipole interactions and electrostatic repulsive force between the microparticles [14]. This means that the interparticle spacing, corresponding to *d* in Equation (1), can be controlled by changing the attractive force, accompanied by the increase in magnetic field strength. Thus, the Bragg reflection wavelength of the CCs can be easily tuned by the external magnetic field strength. Although there have been numerous precedents with regard to the CC structures of magnetite microparticles, the 1D CC structures are more likely assembled by applying a magnetic field according to the review by Chen [14]. However, the synthetic method of monodisperse magnetite microparticles is very limited. In addition, there have been few reports on the control of microparticle diameter. Considering the possibility of applying these magnetite microparticles to magnetically responsive photonic devices, it is highly demanded that a procedure to synthesize them with desired microparticle diameters is found.

In this article, we report on a promising methodology to synthesize monodisperse magnetite microparticles with diameters of 100–200 nm by adjusting the base concentration of the reaction solution. This size-controllable synthesis procedure is very simple. These magnetite microparticles self-organize the CC structures with visible reflection, even in aqueous suspensions, by applying external magnetic field. The reflection peak wavelength of the CCs can be tuned by the external magnetic field strength, microparticle diameter, and microparticle concentration. Such tunability of magnetically responsive CCs would contribute to the technological developments of next-generation photonic devices such as full-color reflective displays and sensors.

## 2. Experimental Section

Anhydrous ferric chloride (FeCl_3_) was obtained from Nacalai Tesque (Kyoto, Japan). Ethylene glycol, anhydrous sodium acetate, and sodium hydroxide (NaOH) were purchased from FUJIFILM Wako Pure Chemical Corporation (Osaka, Japan). Poly(4-stylenesulfonic acid-*co*-maleic acid) sodium salt (PSSMA) with weight average molecular weight of ~2.0 × 10^4^ was obtained from Sigma-Aldrich Japan (Tokyo, Japan). The molar ratio of poly(4-styrenesulfonic acid) unit and poly(maleic acid) unit was 1:1, according to the data sheet of the manufacturer.

Monodisperse magnetite microparticles were prepared by one-pot hydrothermal synthesis using FeCl_3_ as a precursor. In this study, NaOH concentration was varied in the range of 0.28–0.38 M, as shown in Table 1. Here, a typical synthesis procedure of the monodisperse magnetite microparticles at an NaOH concentration of 0.28 M is described as follows (Table 1, sample code: **M-1**).

A mixture of FeCl_3_ (0.52 g, 3.2 mmol), ethylene glycol (32 mL, 0.58 mol), anhydrous sodium acetate (2.4 g, 30 mmol), PSSMA (0.80 g, 4.5 mmol, monomer equivalent), and 120 µL of ultrapure water was vigorously stirred for 20 min at room temperature. After that, NaOH (0.36 g, 12 mmol) was added to this mixture and stirred at room temperature until completely dissolved. The resulting mixture was transferred into a 50 mL Teflon-lined stainless-steel autoclave and heated at 190 °C for 16 h. After cooling down to room temperature, the products were collected by a handheld magnet and washed three times with a mixture of water and ethanol at a volume ratio of 2:1, and finally dried in a vacuum for 24 h.

X-ray diffraction (XRD) patterns of magnetite microparticles were acquired by an X-ray diffractometer (RINT2500, Rigaku, Tokyo, Japan) using Cu Kα radiation under the acceleration voltage of 40 kV. Morphological size and shape of as-prepared magnetite microparticles were characterized by a field-emission scanning electron microscopy (SEM; JSM-7800F Prime, JEOL, Tokyo, Japan) operated at an acceleration voltage of 15 kV. The samples for SEM observation were prepared by depositing the dilute suspensions of magnetite microparticles on a silicon wafer and subsequent drying them at room temperature. Before SEM observation, the sample surface was coated with a thin layer of osmium oxide. Coefficients of variation (CV) in microparticle diameter, which are numerically defined as the ratio of the standard deviation to the average diameter, were calculated from the SEM images. Magnetic properties of magnetite microparticles at 27 °C were measured by using a superconducting quantum interference device (SQUID) magnetometer (MPMS-XL7AC, Quantum Design, San Diego, CA, USA). Zeta potential measurements were conducted using a microparticle charge analysis system (Zetasizer Nano ZS, Malvern Panalytical Ltd., Worcestershire, UK).

Reflection spectra of aqueous suspensions of magnetite microparticles were taken on a compact charge-coupled device spectrometer (USB 2000, Ocean Optics, Orlando, FL, USA) equipped with a halogen light source (HL-2000, Ocean Optics) and an optical fiber with a reflection probe (R200-7-UV-VIS, Ocean Optics). After the aqueous suspension of magnetite microparticles was placed in a quartz glass cuvette with an optical path length of 1.0 cm, the reflection spectra were measured upon applying the external magnetic field, which was generated by an NdFeB magnet with a size of 4 × 4 × 1 cm (NK066, Niroku Seisakusho, Kobe, Japan). The magnetic field strength was tuned by adjusting the distance between the cuvette and the NdFeB magnet and measured using a tesla meter (TM-801, KANETEC Co., Ltd., Nagano, Japan).

## 3. Results and Discussion

### 3.1. XRD Measurements of Magnetite Microparticles

**Figure 1:**
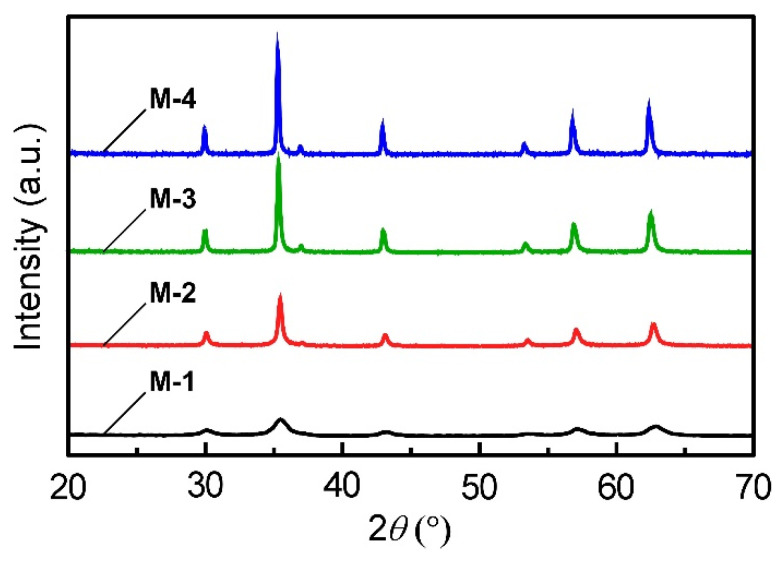
XRD patterns of the magnetite microparticles of **M1**–**M4** with different diameters prepared by the hydrothermal synthesis.

As-prepared colloidal microparticles exhibited intrinsic color of dark brown, arising from light absorption of magnetite. Therefore, we measured XRD patterns of the microparticles in order to characterize their chemical identity. Figure 1 shows XRD patterns of a series of microparticles of **M-1**–**M-4** synthesized at different NaOH concentrations in the hydrothermal synthesis. All the peak positions in each XRD pattern were almost identical, regardless of the difference in synthetic conditions of magnetite microparticles. It is plausible that the peaks at 2*θ* of 30.0°, 35.3°, 36.9°, 42.9°, 53.2°, 56.7°, and 62.3° are assigned to the diffractions of (220), (311), (222), (400), (422), (511), and (440) lattice planes of the face-centered cubic lattices of magnetite, respectively. In addition, these XRD peaks can be indexed to the JCPDS file, No. 19-0629. Therefore, from these XRD results, we concluded that the colloidal microparticles consisting of magnetite can be prepared by the hydrothermal synthesis of FeCl_3_ at NaOH concentrations of 0.28–0.38 M.

### 3.2. Size Control of Magnetite Microparticles

**Figure 2:**
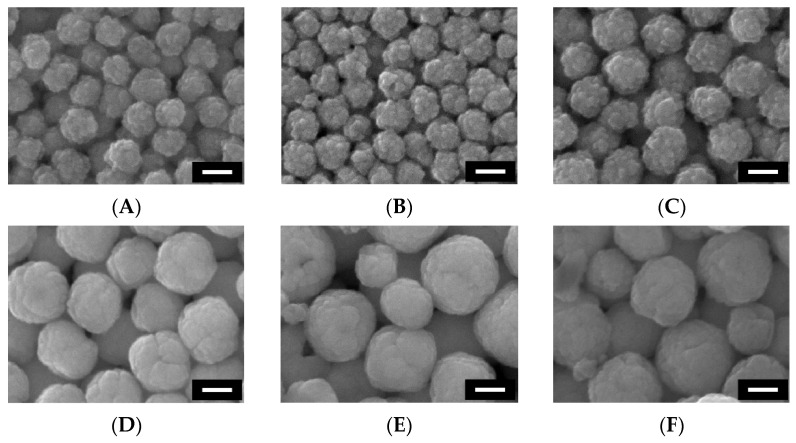
(**A**–**F**) SEM images of a series of magnetite microparticles of **M-1**–**M-6**. All white scale bars represent 100 nm.

Morphological size and shape of the magnetite microparticles were analyzed by SEM observation. The SEM image of **M-1** is shown in Figure 2A. The average diameter and the CV value were determined to be 106 nm and 10.8%, respectively. In general, the monodispersity of microparticle diameter is indispensable to the formation of well-ordered CC structures. For instance, it has been reported that the CV values of colloidal microparticles used for the fabrication of CCs were 5.5% in the case of silica microparticles and 4.2% for PS microparticles in the previous studies [20,21]. By considering the empirical facts, we anticipated that **M-1** can be used for the preparation of CCs owing to its moderately low CV value. However, the detailed SEM observation revealed that the surface of **M-1** is not smooth, in contrast to the conventional colloidal microparticles of silica or PS. This morphological result infers that **M-1** consists of spherically shaped clusters of small magnetite particles with a diameter of several nanometers [22]. The mechanism for the formation of monodisperse magnetite clusters can be explained as follows. The growth process of magnetite microparticles can be divided into two sequential steps according to the previous work by Yu and co-workers [23]. The first step is nucleation, in which primary magnetite nanocrystals (NCs) are formed by the hydrolysis of FeCl_3_. Subsequently, these magnetite NCs aggregate into clusters to reduce their surface energy. Because this growth process of magnetite microparticles, that is, the aggregation of magnetite NCs, selectively occurs inside the PSSMA network, magnetite microparticles with narrow size distribution can be synthesized [24]. From these results, the magnetite microparticles exhibited reasonable monodispersity by the hydrothermal synthesis of FeCl_3_ under this reaction condition.

Subsequently, we attempted to control the magnetite microparticle diameter by changing the reaction conditions. This is because the control of microparticle diameter is of paramount importance from both scientific and technological perspectives of CC structures. Because the Bragg reflection wavelength of CCs is dependent on the lattice constant, which is affected by the difference in microparticle diameter, in this work, another series of magnetite microparticles were synthesized at the different NaOH concentrations from 0.31 M to 0.38 M. These magnetite microparticles, noted as **M-2**, **M-3**, and **M-4** in Table 1, were spherically-shaped clusters of small magnetite microparticles similar to **M-1**, as confirmed from SEM images (Figure 2B–D). Although **M-1** showed 106 nm in the microparticle diameter of, as mentioned above, the average diameters of **M-2**–**M-4**, these increased from 111 nm to 200 nm, accompanied by an increase of NaOH concentrations. However, the CV values were approximately 6–10%, regardless of the NaOH concentrations (Table 1). Therefore, **M-2**–**M-4** were also found to be magnetite microparticles with relative monodispersity. When the NaOH concentration was further increased to 0.44 M, magnetite microparticles also showed ~220 nm in diameter. However, these magnetite microparticles have poor dispersibility in ultrapure water, probably due to the sedimentation caused by the increase in microparticle weights or by the enhancement of van der Waals attractive force induced by the increase in particle diameter. The increase in magnetite microparticle diameter can be explained by the increase in the size of primary NCs. Here, the size of primary NCs (*D*) was estimated by the following Debye–Scherrer equation:

(2) $$D=\frac{0.9\times \lambda }{\beta_{1/2}\times \cos\theta }$$

where *λ* stands for the wavelength of Cu Kα radiation, corresponding to 0.154 nm, *θ* is the diffraction angle, and *β*_1/2_ is the full width at half maximum value of a diffraction peak. In this work, we focused on an outstanding diffraction peak of (311) lattice planes at 2*θ* of 35.3°. By applying the *β*_1/2_ values of (311) diffraction peaks in the XRD patterns into Equation (2), the *D* values of **M-1**, **M-2**, **M-3**, and **M-4** were estimated to be 7.3 nm, 24 nm, 37 nm, and 44 nm, respectively (Table 1). In addition, the diffraction peaks were also intensified owing to the increase in the size of primary NCs. Thus, it can be anticipated that the increase in magnetite microparticle diameter is triggered by the increase in the size of primary NCs. We also considered that the higher alkaline conditions accelerate the hydrolysis of FeCl_3_, which result in the formation of larger primary magnetite NCs.

Finally, we tried to further increase the microparticle diameter by elevating the reaction temperature. Figure 2E,F show the SEM images of **M-5** and **M-6**, which were prepared by the hydrothermal synthesis conducted at 210 °C and 230 °C, respectively. **M-5** and **M-6** were also found to be magnetite microparticles, as confirmed from XRD measurements (Supplementary Materials, Figure S1). However, the diameters of each magnetite microparticle were almost unchanged when compared to **M-4**. Moreover, as evident from SEM images, the CV values of **M-5** and **M-6** deteriorated to around 20%, probably because the nucleate growth of magnetite NCs was uncontrollable. From these results, we found that the appropriate temperature for the synthesis of monodisperse magnetite microparticles is 190 °C and the microparticle diameter can be controlled by changing the NaOH concentration from 0.28 M to 0.38 M.

### 3.3. Water Dispersibility and Magnetic Properties of Magnetite Microparticles

The magnetite microparticles of **M-1**–**M-4** could be easily dispersed in ultrapure water. This was achieved by the removal of excess ions by washing with a mixture of ethanol and water as well as the surface modification of magnetite microparticles with PSSMA. As mentioned in the Experimental Section, PSSMA is a copolymer of poly(*p*-styrenesulfonic acid) and poly(maleic acid). The sulfonic groups in poly(*p*-styrenesulfonic acid) moieties are completely ionized in aqueous solutions while the carboxy groups in poly(maleic acid) moieties can strongly coordinate with iron cations on the magnetite microparticle surface. Thus, the outermost surface of magnetite microparticles is highly negatively charged to offer high dispersibility in water. To evaluate the dispersibility of magnetite microparticles, we measured the zeta potential of **M-3**. As a result, the zeta potential of **M-3** was determined to be −45 mV. This value of zeta potential is comparable to that of monodisperse silica microparticles used for the fabrication of CCs in a previous study [25]. Thus, it can be anticipated that the magnetite microparticles hardly aggregate in water owing to the large electrostatic repulsive force offered by the highly negatively charged surface. By considering these facts, we concluded that the magnetite microparticles are suitable for the fabrication of CCs owing to their monodispersity and high dispersibility in water.

**Figure 3:**
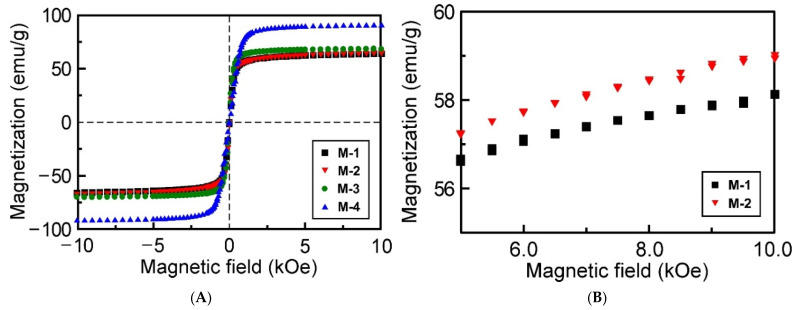
(**A**) Magnetic hysteresis loops of magnetite microparticles measured at 27 °C. (**B**) An enlarged profiles of **M-1** and **M-2** in the range of 5 kOe to 10 kOe for comparison of their magnetization difference.

The magnetic properties of magnetite microparticles of **M-1**–**M-4** were analyzed by SQUID measurements. Figure 3 shows the magnetic hysteresis loops of **M-1**–**M-4** measured at 27 °C. The magnetite microparticles showed no remanence and coercivity, indicative of their superparamagnetic feature. As the external magnetic field increases, the induced magnetization saturates at a certain value, which is so-called saturation magnetization (*M*_s_). From the measurements, the *M*_s_ values of **M-1**, **M-2**, **M-3**, and **M-4** were 58.1 emu/g, 60.5 emu/g, 63.0 emu/g, and 82.3 emu/g, respectively. The *M*_s_ values were monotonously increased in the order of microparticle diameter. The increase in *M*_s_ value can be explained by the large magnetic domains caused by the increase in microparticle diameter. Previously, it has been reported that the *M*_s_ value of polystyrene encapsulating superparamagnetic microparticles used for the fabrication of CCs was 38.6 emu/g, which is much lower than those of **M-1**–**M-4** [26]. Thus, it can be anticipated that these magnetite microparticles of **M-1**–**M-4** can show superior magnetic responsivity when they are applied to CCs.

### 3.4. Optical Properties of Magnetite Microparticle Suspensions

Under an external magnetic field, the magnetite microparticles were quickly self-assembled into CC arrays in fluid aqueous suspensions owing to their highly charged surface, resulting in the appearance of visible Bragg reflection. Monodisperse magnetite microparticles in suspensions are known to spontaneously form the 1D chain-like CC structures under an external magnetic field, which can be formed by the balancing of the interaction of magnetic dipole–dipole attractions and electrostatic repulsive force between microparticles [14]. As the force balance fluctuates by tuning the magnetic field strength, the Bragg reflection wavelength shifts immediately triggered by changes in the spacing between the lattice plane of the 1D chain-like CC structures.

**Figure 4:**
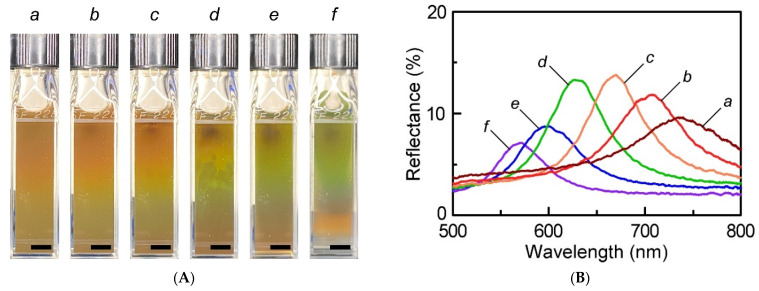
Changes in snapshot image (**A**) and reflection spectrum (**B**) of a suspension of M-3 as a function of the external magnetic field of 28 mT (a), 36 mT (b), 48 mT (c), 65 mT (d), 90.4 mT (e), and 220 mT (f). All black scale bars in the images represent 0.5 cm.

Figure 4A,B show the changes in snapshot image and the reflection spectrum of an aqueous suspension of **M-3** at the microparticle concentration of 0.30 wt%, respectively, upon changing the external magnetic field in a continuous way. The Bragg reflection colors were observed only along the direction of external magnetic field, implying that magnetite microparticles in suspensions form the 1D chain-like CC structures under an external magnetic field. The reflection spectra were measured for magnetite microparticle suspensions placed in a quartz glass cuvette. The external magnetic field was generated by an NdFeB magnet, and its strength was controlled by adjusting the distance between the NdFeB magnet and the cuvette. The reflection color was changed from red to green by moving the magnet at a distance from 4.0 cm to 0.5 cm (Supplementary Materials, Figure S2), corresponding to the external magnetic field from 28 mT to 220 mT, respectively (Figure 4A).

We also observed that the Bragg reflection peak blue-shifts from 730 nm to 570 nm in a continuous way upon increasing the magnetic field (Figure 4B). Such a blue-shift of reflection peak wavelength can be explained as follows. Applying the aqueous suspension of **M-3** at relatively high external magnetic field brings about enhancement in attractive forces between the magnetite microparticles, thereby leading to the geometric decrease in interparticle spacing of the 1D chain-like CC structure. As a result, the Bragg reflection wavelength shifts to shorter wavelengths as the interparticle spacing decreases. Thus, the Bragg reflection wavelength of the 1D chain-like CC structure was found to depend on the external magnetic field strength. Notably, the magnetic response of the Bragg reflection color changes of magnetite microparticle suspension was very quick and fully reversible, as can be seen in the demonstration video (Supplementary Materials, Video S1), which enabled the on-demand tuning of the Bragg reflection peak of the CC by applying with the magnetic field.

**Figure 5:**
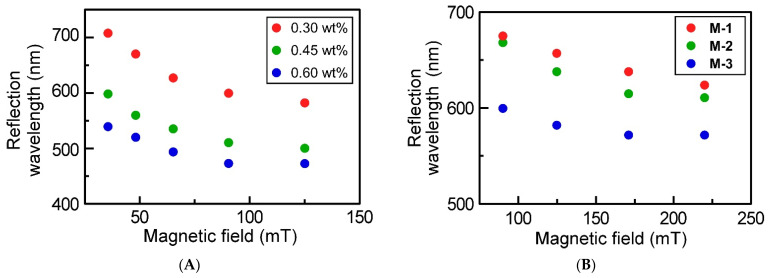
(**A**) Changes in the reflection peak wavelengths of aqueous suspensions of **M-3**, whose concentrations were adjusted to 0.30 wt%, 0.45 wt%, and 0.60 wt%, as a function of the external magnetic field in the range between 36 mT and 125 mT. (**B**) Changes in the reflection peak wavelengths of aqueous suspensions of **M-1**, **M-2**, and **M-3** at same concentration of 0.30 wt% as a function of the external magnetic field in the range between 91 mT to 220 mT.

According to another finding, the Bragg reflection wavelength was also affected by the microparticle concentrations. To demonstrate this ability, three different kinds of suspensions of **M-3** were prepared at the concentrations of 0.30 wt%, 0.45 wt%, and 0.60 wt%. The dependences of the Bragg reflection wavelength on the microparticle concentration are shown in Figure 5A. By comparing the Bragg reflection wavelength under the same magnetic field strength in the range from 36 mT to 125 mT, it turned out that the Bragg reflection wavelength shifts to a shorter wavelength as the microparticle concentration increases. For example, when an external magnetic field with 125 mT was applied to each suspension, the Bragg reflection wavelength shifted from 473 nm to 582 nm by diluting the microparticle concentration from 0.60 wt% to 0.30 wt%. This happened from the geometric decrease in lattice constant between the magnetite microparticles caused by the reduction in the filling ratio of magnetite microparticles [27]. Thus, the Bragg reflection wavelength of 1D chain-like CC structure was found to be dependent on the microparticle concentration in suspension similar to the 3D CC structures composed of non-magnetic spherical microparticles such as silica, PS, and poly(*N*-isopropylacrylamide) hydrogel [13,27].

As addressed in Section 3.2, one-pot hydrothermal synthesis of FeCl_3_ adopted in this study enabled the preparation of magnetite microparticles with a diameter of 106–200 nm. Finally, we attempted to examine the dependence of Bragg reflection wavelength observed for aqueous suspension of magnetite microparticles on the particle diameter. This is because the lattice constant is dependent on the microparticle diameter. However, it is unclear in the case of 1D chain-like CC structures of the magnetite microparticles. To reveal the effect of magnetite microparticle diameters, the suspensions of **M-1**, **M-2**, and **M-3** were prepared. At this time, the concentration of each suspension was fixed to 0.30 wt%. Figure 5B shows the Bragg reflection wavelength of each suspension as a function of magnetic field strength. By comparing the Bragg reflection wavelength under the same strength of the external magnetic field in the range from 90 mT to 220 mT, the Bragg reflection wavelength shifts to shorter wavelengths as the microparticle diameter increases. For example, as applying an external magnetic field of 125 mT, the suspensions of **M-1**, **M-2**, and **M-3** showed the Bragg reflection wavelengths of 657 nm, 638 nm, and 582 nm, respectively. Such a shift of Bragg reflection wavelength can be ascribed to the difference in the *M*_s_ value. The *M*_s_ value increases with the magnetite microparticle diameter, as noted in Table 1. This suggests that larger magnetite microparticles have stronger attractive forces between microparticles, and the reflection wavelength of suspension can shift to shorter wavelengths when an external magnetic field large enough to saturate the magnetization is applied. Therefore, the Bragg reflection wavelength of the 1D chain-like CC structure of the magnetite microparticles was found to depend on the microparticle diameter.

## 4. Conclusions

In this study, we have established a promising methodology to synthesize monodisperse magnetite microparticles with diameters of 100–200 nm by adjusting the base concentration of the reaction solution. By applying an appropriate external magnetic field, the magnetite microparticles were immediately self-assembled into the CC structures, even in fluid aqueous suspensions, owing to their highly charged surface, thereby leading to the emergence of Bragg reflection peak in the visible wavelength range. The Bragg reflection peak could be continuously tuned from 730 nm to 570 nm by the increase in the external magnetic field from 28 mT to 220 mT. Such visible reflection characteristics of CC structures of magnetite microparticles in suspensions were found to depend on the microparticle concentration in suspension and the size of the magnetite microparticles. The present report provides the fine control of microparticle diameter as well as the investigation of these reflection properties of CC structures, which would contribute to the technological developments of full-color refractive displays and sensors by utilizing these monodisperse magnetite microparticles.

## Figures and Tables

**Table 1:** Amounts and concentrations of NaOH in the hydrothermal synthesis and size properties and saturation magnetization (*M*_s_) values of the resultant magnetite microparticles.

Sample	Amount of NaOH (g)	NaOH Concentration (M)	Reaction Temperature (°C)	Diameter (nm) ^1^	CV (%) ^1^	*D*^2^ (nm)	*M*_s_ ^3^ (emu/g)
**M-1**	0.36	0.28	190	106	10.8	7.3	58.1
**M-2**	0.40	0.31	190	111	10.4	24	60.5
**M-3**	0.44	0.34	190	150	6.4	37	63.0
**M-4**	0.48	0.38	190	200	7.0	44	82.3
**M-5**	0.48	0.38	210	226	25.8	– ^4^	– ^4^
**M-6**	0.48	0.38	230	226	18.6	– ^4^	– ^4^

^1^ Determined by SEM observation. ^2^ Determined by Debye–Scherrer equation using the (311) diffraction peak in the XRD pattern. ^3^ Determined by SQUID measurement. ^4^ Not measured.

## Supplementary Materials

The following supporting information can be downloaded at: https://www.mdpi.com/article/10.3390/ma15144943/s1, Figure S1: XRD patterns of the magnetite microparticles of **M-5** and **M-6** prepared by hydrothermal synthesis; Figure S2: Spatial dependence of the magnetic field strength of the NdFeB magnet used in this work; Video S1: Demonstration of reflection color changes observed for a 0.30 wt% aqueous suspension of **M-3** by applying the external magnetic field.
