# Supplementary material for: Size-Controllable Synthesis of Monodisperse Magnetite Microparticles Leading to Magnetically Tunable Colloidal Crystals
Source: Materials (Basel). 2022 Jul 15;15(14):4943. doi: 10.3390/ma15144943 (PMC9323182; doi:10.3390/ma15144943)
Supplement: Supplementary file 1 [file materials-15-04943-s001.zip › materials-1784052-supplementary.pdf]

Size-Controllable Synthesis of Monodisperse Magnetite Microparticles Leading to Magnetically Tunable Colloidal Crystals

Toya Seki, Yutaro Seki, Naoto Iwata and Seiichi Furumi*

Department of Chemistry, Graduate School of Science, Tokyo University of Science, 1-3 Kagurazaka, Shinjuku, Tokyo 162-8601, Japan

*E-mail: furumi@rs.tus.ac.jp.

1. Supplementary Figures

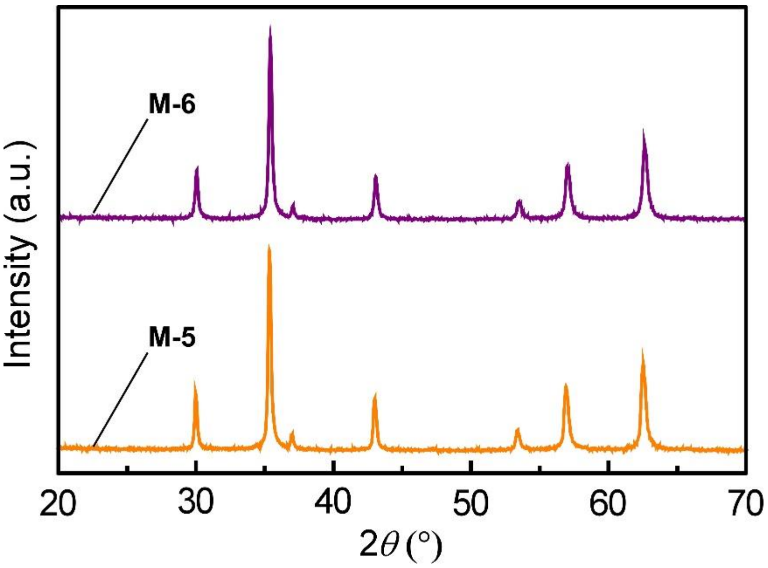

Figure S1. XRD patterns of the magnetite microparticles of M-5 and M-6 prepared by the hydrothermal synthesis.

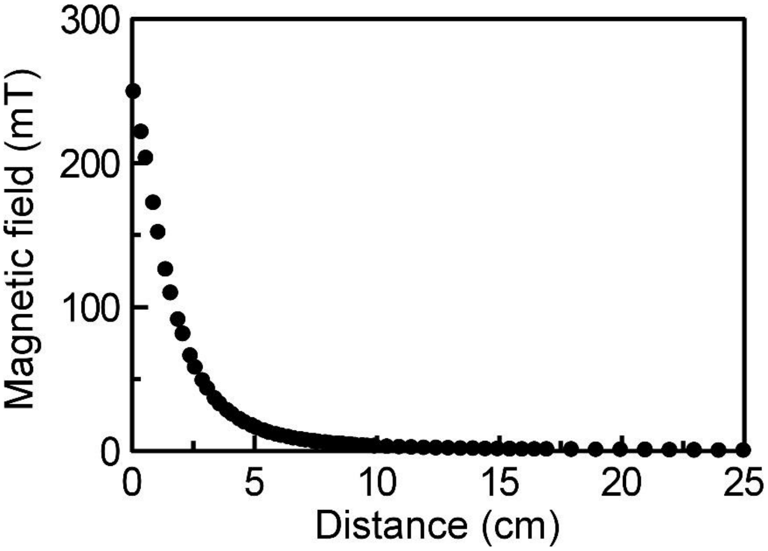

Figure S2. Spatial dependence of the magnetic field strength of the NdFeB magnet used in this work.

2. Supplementary Video

Video S1: Demonstration of reflection color changes, observed for a 0.30 wt% aqueous suspension of **M-3**, by applying the external magnetic field (MP4).
